# Manifold Learning of Dynamic Functional Connectivity Reliably Identifies Functionally Consistent Coupling Patterns in Human Brains

**DOI:** 10.3390/brainsci9110309

**Published:** 2019-11-04

**Authors:** Yuyuan Yang, Lubin Wang, Yu Lei, Yuyang Zhu, Hui Shen

**Affiliations:** 1 College of Intelligence Science and Technology, National University of Defense Technology, Changsha 410073, China; yangyuyuan_nudt@126.com; 2 Institute of Military Cognition and Brain Sciences, Academy of Military Medical Sciences, Beijing 100850, China; wanglb@bmi.ac.cn (L.W.); leiy8805@gmail.com (Y.L.); gfszhuyuyang@yeah.net (Y.Z.)

**Keywords:** manifold learning, consist coupling patterns, resting state, dynamic functional connectivity, sleep deprivation

## Abstract

Most previous work on dynamic functional connectivity (dFC) has focused on analyzing temporal traits of functional connectivity (similar coupling patterns at different timepoints), dividing them into functional connectivity states and detecting their between-group differences. However, the coherent functional connectivity of brain activity among the temporal dynamics of functional connectivity remains unknown. In the study, we applied manifold learning of local linear embedding to explore the consistent coupling patterns (CCPs) that reflect functionally homogeneous regions underlying dFC throughout the entire scanning period. By embedding the whole-brain functional connectivity in a low-dimensional manifold space based on the Human Connectome Project (HCP) resting-state data, we identified ten stable patterns of functional coupling across regions that underpin the temporal evolution of dFC. Moreover, some of these CCPs exhibited significant neurophysiological meaning. Furthermore, we apply this method to HCP rsfMR and tfMRI data as well as sleep-deprivation data and found that the topological organization of these low-dimensional structures has high potential for predicting sleep-deprivation states (classification accuracy of 92.3%) and task types (100% identification for all seven tasks).In summary, this work provides a methodology for distilling coherent low-dimensional functional connectivity structures in complex brain dynamics that play an important role in performing tasks or characterizing specific states of the brain.

## 1. Introduction

The human brain is a hierarchically organized complex system that can be empirically parsed into functionally specialized units commonly referred to as functional brain networks. A large body of neuroimaging studies have made substantial progress in delineating this functional architecture mainly based on resting-state static functional connectivity (sFC). These stable spatiotemporal patterns of resting-state functional activity in a population closely resemble patterns of evoked task-based brain activity [1] and have a significant biological and genetic basis [2,3]. These distributed functional networks cooperate with one another to respond to internal and external stimuli, which underpin various cognitive tasks in the brain. Therefore, identification of the brain’s functional architecture has important significance to understanding information processing procedures in the brain and the relationship between brain functions and individual behavior.

Recently, the use of the functional brain network has been extended from sFC measured based on the entire scan to its dynamic aspect (i.e., dynamic functional connectivity, dFC) that assumes evident time-varying fluctuation of network connectivity during a period of unconstrained rest [4,5,6] and across task states [7,8]. Some generative and computational models were proposed to understand how the functional brain network evolves over time [9]. These studies usually identified consistent and replicable whole-brain connectivity patterns (i.e., transient functional connectivity states) that recur over time under the hypothesis of separate or mixture models of dFC. In the separate model, dFC was expressed by a single spatial connectivity pattern at a given point in time [4,10]. Some studies, however, suggested that a linear mixture model of a smaller number of latent connectivity patterns that recur at varying times may be a better model for dFC [11]. For instance, principal component analysis (PCA) [12] and independent component analysis (ICA) [13] have been used to identify “eigenconnectivities” that reflect meaningful patterns in FC fluctuations assuming the orthogonality or independence of building blocks of network configurations. It is noticeable that many of these previous studies have focused on temporal decomposition of dFC. Few of them, however, concerned the spatial structures underlying dFC evolution.

In this paper, we ask whether there are intrinsic coupling patterns stably existing during the dynamics of functional connectivity, i.e., some functional connections can be grouped into different clusters based on the similarity of their correlation time courses. These clusters, if they exist, would reflect the functionally homogeneous regions that constitute the spatial components supporting dFC evolution. For example, a top-down parcellation strategy using time courses of instantaneous connectivity (a special form of dynamic connectivity) has been confirmed to produce biologically valid functional subdivisions of the cortical cortex and subcortex [14]. It has also been suggested that compared with sFC analysis, functional parcellation based on dFC could exhibit smaller subdivision clusters and significantly improve the reproducibility of segmented subregions across subjects [15]. In contrast to previous time decomposition models of dFC, these studies model time-varying functional connectivity from the perspective of cortical coupling, which was expected to deepen our understanding of the dynamic functional organization of the brain and the temporal evolution of functional brain networks.

In particular, we used manifold learning of local linear embedding (LLE) to extract the consistent connectivity patterns (CCPs) underlying dFC. As a fully data-driven approach, manifold learning has been gradually applied to neuroimaging analysis with many advantages of its shared manifold nature, geometric intuition, and high-dimensional data visualization, including detecting activated voxels [16], reducing dimensionality of fMRI classification [17], generating brain fingerprints [18] and decoding visual stimuli from brain activity [19]. Unlike linear models such as PCA and ICA, the manifold learning of LLE can uncover temporal or spatial structures in observation data as an effective nonlinear dimensionality reduction approach, when optimally preserving the local similarity of data points. In the present study, we assumed that a limited number of whole-brain connectivity patterns stably exist during the evolution of dFC. Furthermore, manifold learning was used to learn the organization of these coupling patterns in high dimensions and to extract the low-dimensionality representation underlying dFC. The Euclidean distance between data points was quantified via the similarity of correlation time courses between connections. An unsupervised classifier based on K-means clustering was then used to separate the spatial components of dFC into CCPs.

We further demonstrated that these CCPs (the embedded dataset structures) have significant neurophysiological relevance by applying the proposed model to discriminate cognitive tasks from the resting state and to detect changes in brain activity induced by sleep deprivation. We assumed that different cognitive tasks or varying brain states could “activate” discrepant CCPs in dFC that have differentiated topological structures. The topological organization of these CCPs was quantitatively evaluated based on one core-quality score with each node (connection). We further demonstrated the potential of the CCPs’ topological organization for classifying the sleep-deprivation condition and different cognitive tasks with high accuracy, suggesting that these coherent low-dimensional functional coupling structures in complex brain dynamics play an important role in performing tasks or characterizing specific states of the brain.

## 2. Materials and Methods

### 2.1. Participants

The primary dataset used in this work was selected from the Q3 release of the Human Connectome Project dataset (HCP). A second dataset (the sleep-deprivation dataset) was used to verify the effects of the proposed framework. These details are described below.

**Figure A1:**
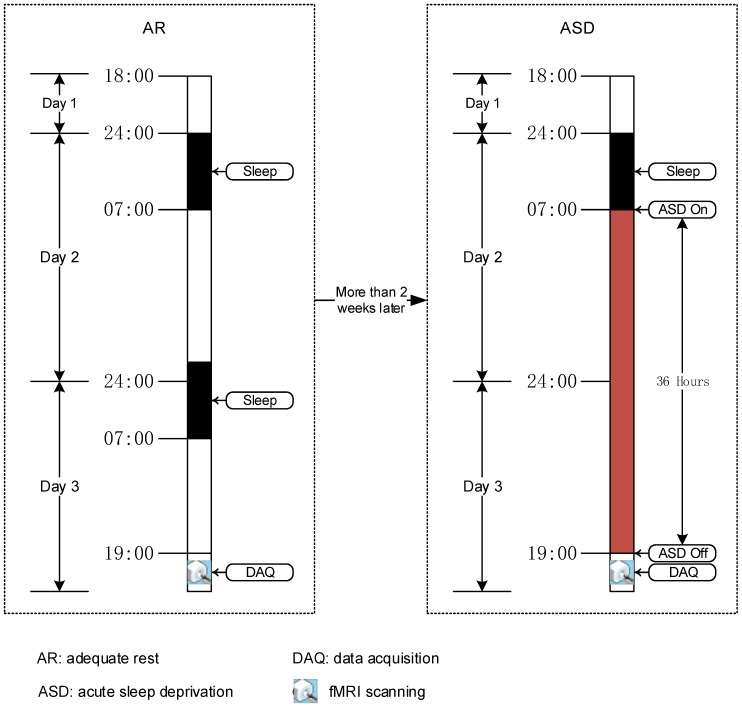
Experimental design and implementation. The participants were scanned twice by the nuclear magnetic resonance spectrometer, and the scans were separated by an interval of more than 2 weeks. During the AR session, participants kept their regular sleep habits and underwent the AR scan from 7:00 P.M. to 8:00 P.M. During the ASD session, participants were required to remain awake and were monitored in the lab from 7:00 A.M. to 7:00 P.M. the next day to make sure that approximately 36 h of total sleep deprivation was accumulated. The two experimental sessions were counterbalanced for all participants who were required to be awake during data acquisition.

(1) HCP dataset: The first dataset consisted of 40 healthy subjects (age: 22–25 years, 5; 26–30 years, 15; 31–35 years, 20; gender: male, 19; female, 21) were selected from the Q3 release of the HCP dataset with low mean head motion (<0.23 mm) during both resting-state sessions [20], including resting-state fMRI (rsfMRI) and seven-task fMRI (tfMRI) data. The resting-state fMRI data for all subjects were scanned in separate sessions on two different days. For the two sessions for each subject, only data from the left-right (LR) phase-encoding run for session 1 were used. rsfMRI data acquisition was performed on a 3.0 T Siemens scanner with the participants’ eyes open, relaxed, and fixed on a bright crosshair projected on a dark background. The data acquisition parameters were as follows: $repetition\;time/echo\;time\left(TR/TE\right)=720\;\mathrm{ms}/33.1\;\mathrm{ms}$, $flip\;angle=52^{\circ }$, $field\;of\;view\;\left(FOV\right)=208\;\mathrm{mm}\times 180\;\mathrm{mm}\left(\mathrm{RO}\times \mathrm{PE}\right)$, $matrix\;size=104\times 90\left(RO\times PE\right)$, resolution=2.0mm, slices=72, and volumes=1200. Please refer to the previous literature for a detailed description of the HCP data and scanning protocol [21]. tfMRI data were acquired with the same EPI pulse sequence parameters as rsfMRI [22]. The time lengths of each task and resting state are as follows: resting state (1200 frames), working memory (405 frames), gambling (253 frames), motor (284 frames), language (316 frames), social cognition (274 frames), relational processing (232 frames), emotional processing (176 frames). The fMRI paradigm structures were controlled for the 7 tasks in the HCP dataset e.g., the working memory task contains 8 task blocks (10 trials of 2.5 s each, for 25 s) and 4 fixation blocks (15 s), and in the motor task, each block of a movement type lasted 12 s. The block duration and block cycle are different in the 7 tasks (all details shown in HCP S1200 Release Reference Manual: https://www.humanconnectome.org).

(2) Sleep-deprivation (SD) dataset: The second dataset comes from the 36 h Acute Sleep Deprivation (ASD) study of 37 healthy, right-handed adult men (age: Mean±STD, 23.1±1.9 years) that was reported in our previous studies [23,24]. All participants had good sleep habits (got to bed no later than 12:00 p.m. and got up at no later than 8:00 a.m.) and slept at least 6.5 h (average: 7.5±0.7 h) every night for the past month. Exclusion criteria were central and peripheral nervous system disease, head trauma, cardiovascular disease and/or hypertension, cataract and/or glaucoma, pulmonary problems, alcohol or drug abuse. All subjects were required to maintain a regular sleep schedule and to avoid alcohol, caffeine, chocolate and napping one week before and during the study. This study was approved by the Research Ethics Committee of Beijing Military General Hospital. Written informed consent according to the Declaration of Helsinki was obtained from all participants. The experimental paradigm is shown in Figure A1 (refer to the previous report [24] for detail). rsfMRI data were acquired using a GE 3.0T Discovery 750 scanner with an 8-channel head coil, and functional images were collected using a gradient echo-planar imaging sequence. The imaging parameters were as follows: repetitiontime(TR)=2000ms; echotime(TE)=30ms; fieldofview(FOV)=240×240mm; slicethickness=3mm; slicegap=1mm; $flip\;angle\left(FA\right)=90^{\circ }$; matrixsize=64×64; and 35 oblique slices. Each resting-state scan lasted 8 min and 240 volumes were collected for each participant.

### 2.2. Data Preprocessing

For the HCP dataset, we adopted the publicly released ICA-FIX denoised fMRI data and used an automated component classifier, referred to as FIX (FMRIB’s ICA-based X-noisifier), to remove non-neural spatiotemporal components from the data after the minimal preprocessing pipelines for HCP functional data which mainly includes spatial artifact and distortion removal, motion correction, within-subject cross-modal registration, and cross-subject registration to a standard space [25]. In addition to the denoised fMRI data, several additional preprocessing steps were performed, including skipping the first 15 volumes, resampling data into 3mm×3mm×3mm, spatial smoothing using a Gaussian filter kernel with 6 mm full-width at half-maximum (FWHM), temporal band-pass filtering [0.01,0.08] Hz, and nuisance regression. Finally, regression of head motion, the white matter (WM) signal, cerebrospinal fluid (CSF) signal and their first-order deviations were performed to reduce spurious variance that was unlikely to reflect neural activity [20]. For tfMRI data, the preprocessing pipelines included motion correction, spatial smoothing, temporal pre-whitening, slice time correction, and global drift removal [21].

SPM12 software (http://www.fil.ion.ucl.ac.uk/spm) was used for preprocessing rsfMRI ASD data. For each participant, the first 5 volumes of scan data were removed for magnetic saturation. The remaining 235 volumes were recalibrated by registering and re-slicing for head movements. Next, normalization to the EPI template in the Montreal Neurological Institute (MNI) space was conducted, and the resulting volumes were resampled to 3mm×3mm×3mm. Then, the normalized images were spatially smoothed with a 6 mm FWHM Gaussian kernel filter and temporally filtered using a Chebyshev band-pass filter (0.01–0.08 Hz). Finally, after the linear trend was eliminated, multiple regression analysis was performed on the data. Additionally, the average signals of head motion, white matter (WM), and cerebrospinal fluid (CSF) and their first-order derivatives were eliminated. Due to several confounding factors such as excessive head motion, potential resilience to ASD and vigilance loss during scanning in the ASD condition, 11 subjects were removed during data acquisition [24]. The remaining 26 qualified subjects’ data were selected to explore the time-invariant FCPs changed by ASD underlying dFC.

### 2.3. Regions of Interest (ROIs)

We used the 160 ROIs atlas previously reported [26] to parameterize whole-brain activity (radius=4mm). These ROIs were further divided into six principal functional networks, including the cerebellum network (CB), cingulo-opercular network (CON), default mode network (DMN), fronto-parietal network (FPN), occipital network (OCN) and sensorimotor network (SMN). Specifically, 27 voxels were included in one ROI, which are the centroid of the ROI and those gray matter voxels located outside ROIs were excluded. The representative signal of each ROI was calculated based on the mean of the internal voxels within this ROI [27].

### 2.4. Estimation of Dynamic Functional Connectivity

**Figure 1:**
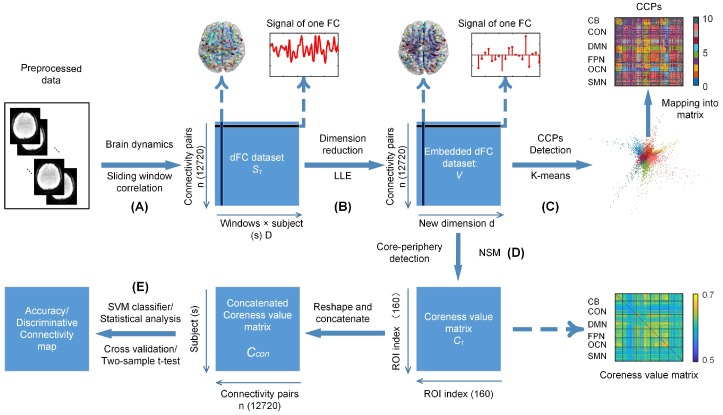
The framework of the manifold learning model. (**A**) Using the sliding-window correlation approach to estimate the dynamic functional connectivity for the participants. The obtained dFC dataset was rearranged into the matrix. The rows of the matrix are the sliding-window signals of the connectivity pairs. The columns of the matrix are all connectivity pairs ordered by the correlation sequence of the ROI atlas. (**B**) Embedding the dataset in a low-dimensional manifold space with the LLE algorithm. The columns of the embedded dFC dataset are mapped into d dimensions. (**C**) Definition of the CCPs for the structure of dynamic functional connectivity. The application of the k-means algorithm separates the manifold structure into d CCPs. We labeled every CCP in one color. (**D**) Measurement of typological organization of the CCPs (dataset). The NSM algorithm for the CCPs assigned every connectivity pair with a nonnegative value such that a larger value indicated a higher level of importance. Then, the value vector was realigned into the ROI-ROI index matrix. (**E**) Classification or statistical analysis for the manifold structure of the dataset. We used the coreness value as the feature to make LOOCV or a 10-fold cross-validation strategy at an individual level, to perform a two-sample *t*-test on a group level and to conduct permutation testing under the null hypothesis for the ASD/AR group.

A sliding-window correlation approach was applied to characterize dynamic functional connectivity (dFC) [4], which had been proven to be a high efficacy framewise dFC method in the numerous dFC estimation methodologies [28]. The temporal window, parameterized by its window size, was shifted by a slide step along a set of timecourses which were extracted from the fMRI images using the 160 ROIs. The timecourse correlations within each window between paired ROIs were obtained with Pearson’s correlation analysis (Figure 1A). A 160×160 real symmetric correlation matrix was obtained for one window and the upper triangle of the matrix was taken and expanded to a 12,720 × 1 vector as the functional connectivity vector of this window. A Fisher-z-transformation was then applied to the vector to ensure the normal distribution of the functional connectivity. Consequently, the dFC matrix was $S=\left\{s_{1},\cdots ,s_{n}\right\}\in R^{n\times D}$, and $s_{i}\in R^{D}$ was the sliding-window signal for *i*th connectivity pair, where *n* (12,720) is the number of the whole-brain functional connectivity pairs and *D* is the number of sliding windows. In this study, the slide step was selected as 5 TRs and the window size was 40 s, which were thought to achieve a balance between the ability to resolve the dynamics and quality of the covariance matrix estimation [4,27].

### 2.5. Manifold Learning of dFC

A manifold learning approach of local linear embedding (LLE) [29] was used to extract the functionally homogeneous connectivity patterns (i.e., CCPs) underlying dFC (Figure 1B). We seek this parametrization of the dataset to obtain a low-dimensional representation of dFC when optimally preserving the local similarity between a windowed time series of connections. The embedding dimensionality of the feature space is a crucial parameter to the manifold learning. The maximum likelihood estimation [30] (MLE) was applied to calculate the intrinsic dimensionality of *S*. For an observation $s_{i}$, the equation executed the MLE dimensionality estimator as

(1) $$\begin{array}{c} {\hat{d}}_{k}\left(s_{i}\right)={\left[\frac{1}{k-1}\sum_{j=1}^{k-1}\log\frac{T_{k}\left(s_{i}\right)}{T_{j}\left(s_{i}\right)}\right]}^{-1}\;. \end{array}$$

where $T_{j}\left(s_{i}\right)$ was the Euclidean distance from $s_{i}$ to its jth nearest neighbors, and $T_{k}\left(s_{i}\right)$ was the sum of $T_{j}\left(s_{i}\right)$. To reduce the influence of manifold fluctuation and noise, the intrinsic dimensionality of the dataset *s* was calculated as the average of ${\hat{d}}_{k}\left(s_{i}\right)$.

(2) $$\begin{array}{c} {\hat{d}}_{k}\left(s\right)=\frac{1}{n}\sum_{i=1}^{N}{\hat{d}}_{s}\left(s_{i}\right)\;. \end{array}$$

We submitted the dFC matrix *S* to the LLE algorithm as the input which includes three steps of transforming the matrix *S* in *D*-dimensional Euclidean space into the embedding matrix *V* in *d*-dimensional manifold space. Step 1: for the sample point $s_{i}$ (connectivity pair), we used Euclidean distance to find its *k* nearest neighbors $s_{j}\left(j=1,2,\cdots ,k\right)$ with the K-nearest neighborhood (KNN) algorithm and enforced that $w_{ij}=0$ if $s_{j}$ did not belong to the set of neighbors of $s_{i}$ and that the rows of the weights matrix *W* summed to one. Step 2: we obtained the optimal reconstruction linear coefficients W by resolving the minimum value of the cost function Equation (3). The constrained weights that minimize these reconstruction errors $\varepsilon \left(W\right)$ obey an important symmetry: for any particular data point, they are invariant to rotations, rescalings, and translations of that data point and its neighbors. By symmetry, it follows that the reconstruction weights $w_{ij}$ characterize the intrinsic geometric properties of each neighborhood, as opposed to properties that depend on a particular frame of reference. Please note that the invariance to translations is specifically enforced by the sum-to-one constraint on the rows of the weight matrix.

(3) $$\begin{array}{c} \varepsilon \left(W\right)=\sum_{i=1}^{n}{∥s_{i}-\sum_{j=1}^{k}w_{ij}s_{j}∥}^{2},\;with\;\sum_{j=1}^{k}w_{ij}=1\;. \end{array}$$

Step 3: the optimal *d*-dimensional coordinates $v_{i}$ for point $s_{i}$ were selected to obtain the minimum value of the cost function $\Psi \left(V\right)$ in embedding space, where

(4) $$\begin{array}{c} \Psi \left(V\right)=\sum_{i=1}^{n}{∥v_{i}-\sum_{j=1}^{k}w_{ij}v_{j}∥}^{2},\;with\;\sum_{i=1}^{n}v_{i}=0,\;\sum_{i=1}^{n}v_{i}v_{i}^{T}=nI_{d\times d}\;. \end{array}$$

This cost function, as with the previous one, is based on locally linear reconstruction errors, but here we fix the weights $w_{ij}$ while optimizing the coordinates $v_{i}$. The embedding cost in Equation (2) defines a quadratic form in the vectors $v_{i}$. Subject to the constraints that make the problem well-posed, it can be minimized by solving a sparse N×N eigenvalue problem, whose bottom d non-zero eigenvectors provide an ordered set of orthogonal coordinates centered on the origin. Thus, we obtained the low-dimensional parameterization *V* of the dFC matrix *S* in manifold space.

### 2.6. Identifying CCPs from HCP rsfMRI Data

The low-dimensional parameterization of the dataset had a specific shape with extended branches, which was expected in the embedding structure with the LLE algorithm. The coordinates of the embedding spaces with the LLE algorithm are related to meaningful attributes: representative faces are shown next to the out-stretching points in the face image experiments and the intersection of two regions locates words with similar contexts in this continuous semantic space in the trial of the semantic associations of words [29]. Therefore, we should pay attention to the out-stretching “arms” and note the correlation of these discrete points and aggregation groups. These distributions might represent the relation of the specialization and cooperation of the connectivity pairs underlying the captured brain fluctuations. Thus, we used the K-means algorithm to differentiate the functionally special and coherent structures. Here, we used a K-means algorithm with similarity mensuration. The distance between two points was calculated by the cosine of their angle to the center of dataset (the origin) to separate these “arms” into the CCPs (Figure 1C). The number of clusters equaled the dimension *d* of the embedding, according to the previous study for the low-dimensional embedding of fMRI dataset, where it was proposed that each dimensional eigenvector would contribute to an independent CCP (cluster) [16]. Then, we conducted the K-means cluster procedure. The *d* clusters were obtained. We displayed these clusters in the functional connectivity map and found the functionally consistent coupling patterns (CCPs). Thus, we defined these clusters with a new name “CCPs”. The *d* CCPs were therefore obtained.

The identified CCPs were reranked based on their spatial overlap rate (SOR), which reflects the spatial correspondence between the CCPs identified from the K-means results of LLE embedding models, i.e., the two CCPs respectively obtained from the HCP dataset and AR group of the sleep models with the highest SOR were more likely to spatially correspond. Specifically, the SOR between the *i*-th CCP for one subject manifold structure model and *j*-th CCP for another subject manifold structure model are calculated using the following equation:

(5) $$\begin{array}{c} SOR_{\left(ij\right)}=\frac{SP_{within\left(ij\right)}}{SP_{total\left(ij\right)}}\;. \end{array}$$

where $SP_{within\left(ij\right)}$ is the number of connectivity pairs simultaneously belonging to the *i*-th CCP from one subject model and the *j*-th CCP from another subject model during the scanning, and $SP_{total\left(ij\right)}$ is the number of connectivity pairs belonging to the *i*-th CCP from the one subject model or the *j*-th CCP from another subject model.

### 2.7. Measuring Topological Organization of CCPs Based on Core-periphery Detection

Spontaneous fluctuation in functional connectivity has been linked to changes in cognitive or vigilance states [31,32], and we hence hypothesize that different cognitive tasks or vigilance levels change the embedding structure in dFC into a discrepant topology. In the embedding of the dFC dataset, the structure between a data point and its neighbors might change the rotation. Thus, the coordinates could be regarded as a hub sign for a manifold structure. We need a method to measure manifold structure in different coordinate systems.

Here, we quantified the manifold structure of CCPs (embedding dataset) by computing a core-quality value [33] for each node (connection) of the embedding dataset *V*. Core-periphery detection was performed by using a nonlinear spectral (NSM) algorithm [33] derived from work on the nonlinear Perron-Frobenius theory [34,35,36]. This approach assigns a nonnegative value to each node such that a smaller value indicates a lower level of importance of that node in the manifold structure. The key point was to determine a core-periphery ranking vector x>0 that assigned to each connection a distinct positive number between 1 and *n*, with a higher rank denoting a more core connection. The core-periphery profile function $\gamma \left(x\right)$ is defined as

(6) $$\begin{array}{c} \gamma \left(x\right)=\frac{\sum_{i,j=1}^{k}A_{\pi_{i},\pi_{j}}}{\sum_{i=1}^{k}\sum_{j=1}^{n}A_{\pi_{i},\pi_{j}}}\; \end{array}$$

where adjacency matrix *A*, which could be obtained via calculating the embedded representation *V* of the dFC dataset with the KNN algorithm and π is a permutation such that $x_{\pi_{1}}\leq \cdots \leq x_{\pi_{n}}$. In other words, if for each *k*, we regard $\pi_{1},\pi_{2}\cdots ,\pi_{k}$ as peripheral nodes and $\pi_{k+1},\pi_{k+2},\cdots ,\pi_{n}$ as core nodes, then $\gamma \left(x\right)$ measures the ratio of periphery-periphery links to periphery-all links. Hence, *x* reveals a strong core-periphery structure if $\gamma {\left(x\right)}_{k}$ remains small for large *k*. The value *x* varies per iteration of the NSM algorithm until $∥x_{k}-x_{k+1}∥/\left|x_{k+1}\right|<tolerance$ [33]. Thus, the obtained core-periphery value vector $c=x_{k+1}/\max\left(x_{k+1}\right)$ was used to depict the topological organization of the CCPs for each participant (Figure 1D). This core-periphery approach has been proven to outperform previous core-quality function optimization methods: Degree, Perron, and Sim-Ann algorithm proposed in [37].

Herein, the spatial similarity between the core-quality value FC maps was calculated using $eta^{2}$ [38] as the following equation:

(7) $$\begin{array}{c} eta^{2}=1-\frac{CC_{\mathrm{Within}\;}}{CC_{\mathrm{Total}}}=1-\frac{\sum_{i=1}^{v}\left[{\left(a_{i}-m_{i}\right)}^{2}+{\left(b_{i}-m_{i}\right)}^{2}\right]}{\sum_{i=1}^{v}\left[{\left(a_{i}-\bar{M}\right)}^{2}+{\left(b_{i}-\bar{M}\right)}^{2}\right]}\;. \end{array}$$

where $a_{i}$ and $b_{i}$ are the values at position *i* of matrix $C_{a}$ and matrix $C_{b}$, respectively, is the mean of $a_{i}$ and $b_{i}$, and $\bar{M}$ is the total mean value across all positions in the two matrices.

### 2.8. Classifying the HCP Tasks Based on Topology of CCPs

To exemplify the high potential of CCPs’ topological properties from providing specialized whole-brain configuration information at the single-participant level, we implemented the HCP task classification experiment (Figure 1E). First, manifold learning of the dFC matrix was performed for each subject to obtain low-dimensional embedding of dynamic connectivity structures on an individual level. Secondly, we estimated the resting-state and seven-task core-periphery values *c* for each subject as input features. To achieve good prediction, constructing the feature space for the classification model was important. The discriminative power of a feature can be quantized by the value of the two-sample *t*-test for the training dataset. A smaller value means a more discriminative power. For every training and testing loop, feature selection was conducted with the statistical results from the training dataset. Then, the prediction would be performed in the feature selection on the training dataset. Then, we used a linear support vector machine (SVM) classifier to validate the discriminative capacity of the CCPs topology for classifying cognitive task scans from the resting-state scans. For each of the seven classifiers, a leave-one-out cross-validation procedure was performed 100 times to estimate the generalization ability. Finally, the unbiased classification accuracy (ACC), sensitivity (SS) and specificity (SC) were computed based on the results of cross-validation [17]. To justify the model’s usage, the multiple tasks classifier was designed, which tried to identify a single task for the other 6 tasks and rest with a one-versus-the-rest strategy.

### 2.9. Detecting CCP Changes Induced by Sleep Deprivation

We further detected the changes in CCPs induced by sleep deprivation to link the CCP topological properties (core-periphery values) into potential neurophysiological sources. We provided results of two experiments, including an individual prediction for sleep deprivation and a statistical comparison between sleep deprivation (SD) and resting-wake (AR) conditions (Figure 1E). In the first experiment, an individual core-periphery value vector was computed as a classification feature for each of the 26 participants with sleep deprivation. A linear SVM classifier with a 10-fold cross-validation or a leave-one-out cross-validation strategy were used to predict the ASD/AR states of each participant according to their CCPs’ topological organization. In the second experiment, a paired two-sample *t*-test (p<0.05andFWERq<0.05) was performed on the core-periphery value for statistical analysis of sleep deprivation on a group level. The identified statistical significance in CCP topology was suggested to reflect the underlying patterns of brain connectivity that are putatively responsible for neurophysiological changes in the ASD brains. To further validate the results of statistical contrast, we assessed the statistical significance of the changed core-periphery values in the sleep-deprivation condition using permutation testing (Figure 1E) designed to estimate the empirical cumulative distribution of the between-group differences under the null hypothesis of exchangeability. For all core-periphery value vectors, the class labels (e.g., ASD vs. AR) were randomly permuted 1000 times, and the p-values reported in the permutation tests represent the probability of observing the reported differences of core-periphery values by chance.

## 3. Results

### 3.1. The Resting-State CCPs

**Figure 2:**
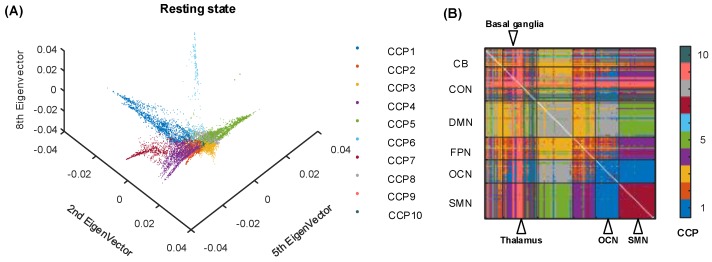
The resting-state CCPs distilled by our method framework. (**A**) Each CCP marked in the same color is distributed in one specific direction. (**B**) The CCPs are rearranged into the ROI-ROI index matrix.

**Figure A2:**
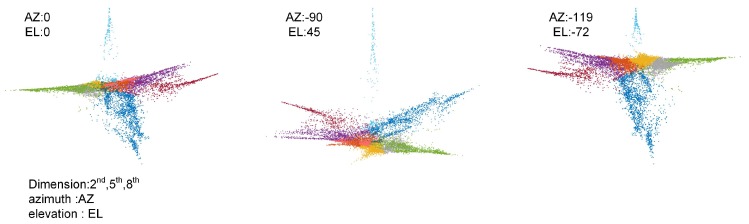
The 3d plot of the manifold from different angles for the 10 CCPs in Figure 2A.

We found that local linear embedding of dFC based on the HCP resting-state dataset leads to a prominent manifold structure of a “specific shape with extended branches” (Figure 2), in which each branch represents the CCP of the resting-state dFC. Therefore, the manifold structure extracted by the LLE algorithm forms the 10 CCPs, which reveal the intrinsic brain organization underlying dFC. To give a better sense of the 10 CCPs’ locations, we displayed the manifold structure from different angles in Figure A2 and from different 3 dimensions in Figure A5. The criterion used for choosing the dimensions in the plot is for a better visualization. The different CCPs are distributed on different “arms”, and the connectivity nodes in the manifold space are plotted with its 2^nd^, 5^th^ and 8^th^ dimension in Figure 2A. Furthermore, all the CCPs were mapped into the correlation matrix of six intrinsic brain networks (Figure 2B). Interestingly, the majority of these resting-state CCPs exhibited significant network-specific coupling patterns. For instance, CCP1 covers almost all connectivity pairs between the OCN and the SMN and within the OCN. The connectivity within the SMN was fully represented by CCP7. In addition to the above network-level CCPs, some CCPs revealed subcortex-cortical connectivity patterns, such as the basal ganglia, thalamus in CCP9 and several functional couplings between a few specific regions of the CON and the whole-brain in CCP10, including the post-insula and temporal, superior temporal, and angular gyri. We estimated the overlap relationship of the CCPs for the embedding representation between the resting-state of the HCP dataset and the AR group in the sleep-deprivation dataset (Table A4). We calculated the core-periphery features for the 10 CCPs (Table A5).

We also directly explored relations between the connectivity pairs of the dFC dataset and the linearly (PCA) embedded dataset. Using the Euclidean distance among the connectivity pairs with a K-means approach, the CCPs in a high-dimensional space were obtained (Figure A4). The high-dimensional CCPs are significantly different from those in the manifold space (Figure 2B) in terms of the aggregation and the basic neural circuit patterns of the same CCPs. Furthermore, the CCPs in a linearly (PCA) embedded space were identified by the Euclidean distance among the samples with the K-means algorithm in Figure A6. The CCPs in Figure 2B obtained from the LLE method have the highest modularity in the functional connectivity map among the naive connectivity CCPs in Figure A4 and the linearly (PCA) embedded CCPs in Figure A6. The PCA embedded CCPs are almost same as the naive connectivity CCPs. In addition, we estimated the matching relationship of the CCPs for the embedding representation among the single subject. The SORs of the CCPs in the manifold space showed little similarity, e.g., subject 1 and subject 2 (Table A2). Then, the core-quality score of the core-periphery structure also showed 50∼65% $eta^{2}$ similarity on an individual level (Due to the paper size limitations, we only show the first 10 people in Table A3).

### 3.2. Different Tasks “Activate" Differentiated Core-Periphery Structure of the CCPs

**Figure 3:**
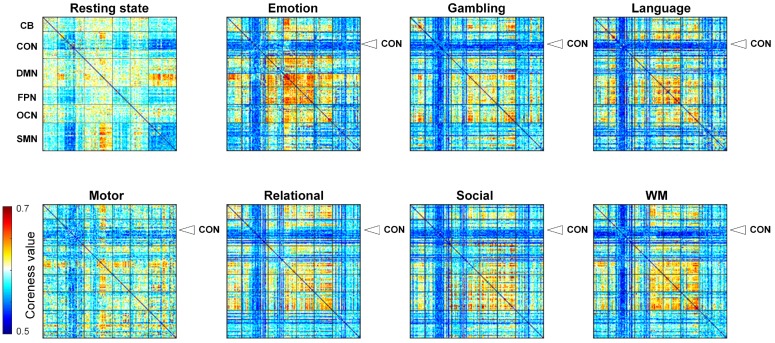
The resting-state and task core-quality value matrix. The coreness vectors of all the connectivity pairs are rearranged into the ROI-ROI index matrix.

More important connectivity in the core-quality value matrix was assigned a larger value within all the connectivity. The NSM algorithm assigned the node (connectivity) core-quality values with the core-quality value vector *c* and we rearranged the core-quality vector *c* into the core-quality matrix *C* using the ROI-based connectivity index in the brain template. Then, the rows and columns of the matrix *C* represent the ROI index in the brain template. After rearrangement using this rule, the elements in the matrix *C* quantify the importance of the functional connectivity in the whole scanning procession between ROIs. Consequently, the functional connectivity NSM value matrices for the resting state and 7 tasks were obtained and shown in Figure 3. The important functional connectivity nodes can be directly perceived in the matrices. In the resting state, nearly all the connectivity nodes show an even distribution level. However, the connectivity between the cingulo-opercular network and sensorimotor network expresses the obvious peripheral influence on the connectivity. With clear distinctions from the resting state, the cingulo-opercular network in all the 7 tasks’ core-quality matrices presents similar peripheralization and displays the lowest interaction with other networks. Meanwhile, the ROI-based connectivity among the default network, fronto-parietal network and occipital network has a larger core impact. If we look at the 7 tasks’ core-quality matrix in detail, the core-periphery quality configuration is unique in one particular task. For example, the network interaction with the default network and sensorimotor network represents a more key impact in the motor task.

**Figure A3:**
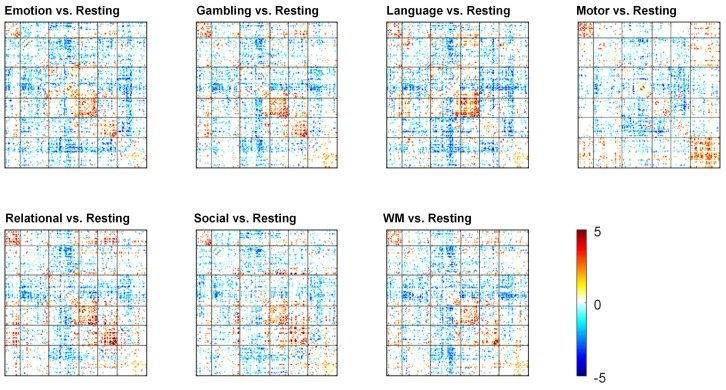
The detailed t values of the distinguished connections for each pair of brain states.

**Figure A4:**
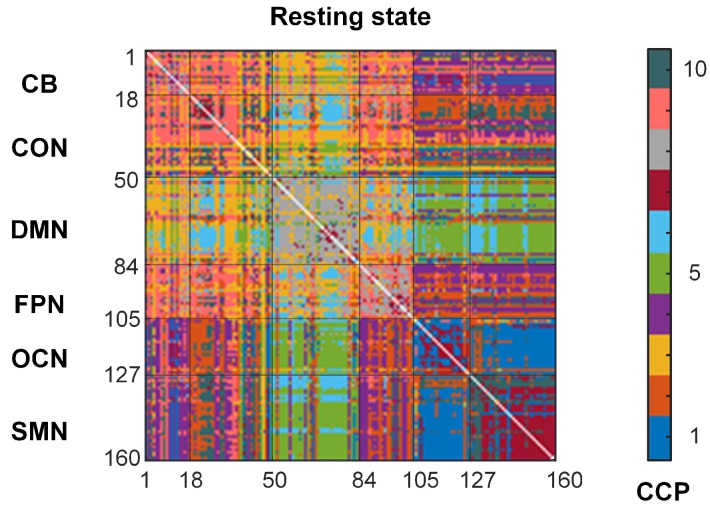
The CCPs in the original Euclidean space obtained with a K-means clustering approach.

**Figure A5:**
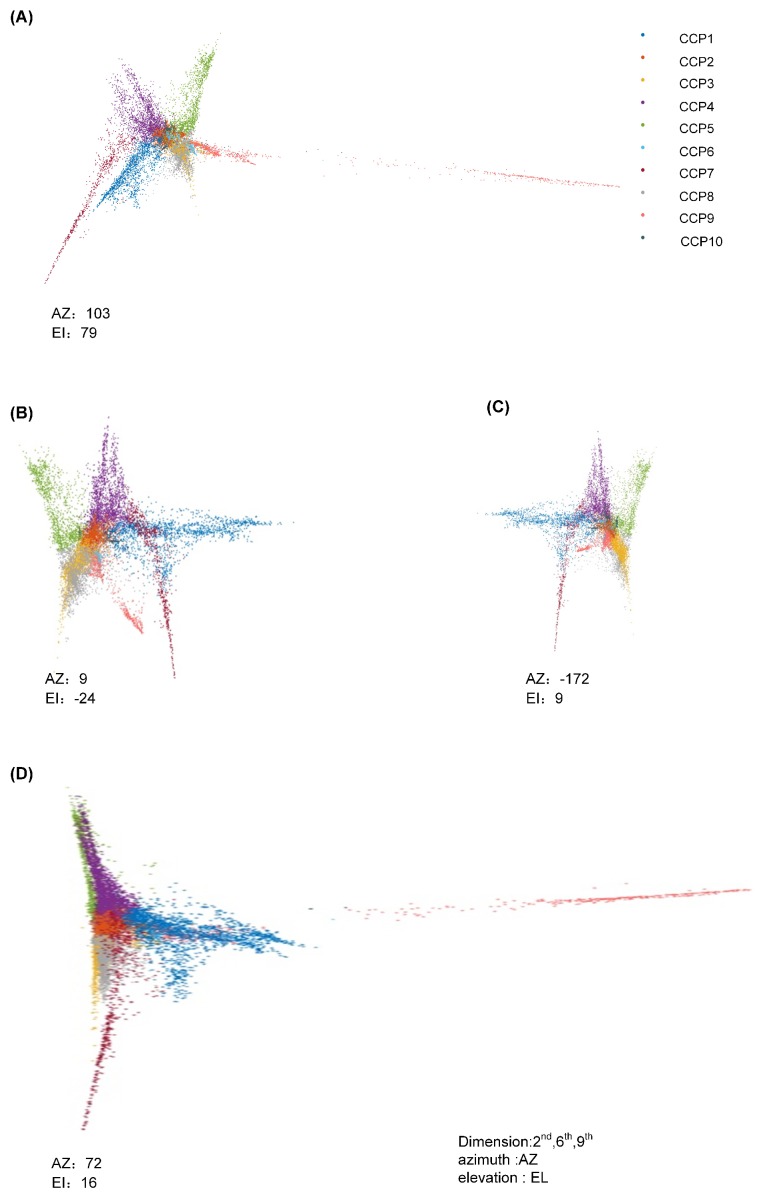
The 3D plot of the manifold viewed from different angles for the 10 CCPs in the $2^{nd},6^{th},9^{th}$ dimension.

**Figure A6:**
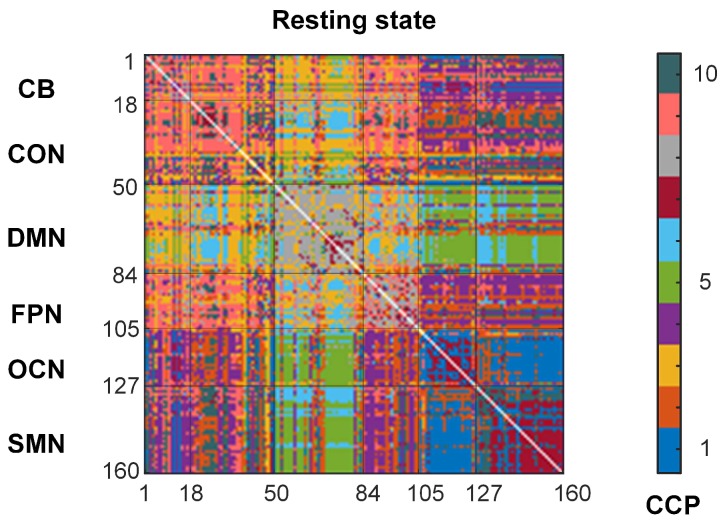
The CCPs in the linearly embedded Euclidean space obtained with a K-means clustering approach.

In addition to estimating the topological organization of CCPs (the core-quality values in manifold structure) with the NSM algorithm, we also assigned the core-periphery values to the connectivity pairs of the dFC dataset (the core-quality values in dFC structure) and the topological organization of the linear structure (PCA dimension reduction) for the resting state and seven tasks. The SVM classifier results showed unbiased classification accuracy (ACC), sensitivity (SS) and specificity (SC) in Table 1 and Table 2. It was noteworthy that the performance of the SVM classifier based on the PCA framework was the lowest (shown in Table A6). The numbers of distinguished connections were 2491, 2156, 2584, 1752, 2141, 2086 and 2102. The detailed *t* values of the distinguished connections for each pair of brain states (shown in Table 1) could be found in Figure A3. The discriminative features are mostly grouped in the network interactions for the prediction between rest and tasks.

### 3.3. The Impact of Acute Sleep Deprivation (ASD) on the CCPs

**Figure 4:**
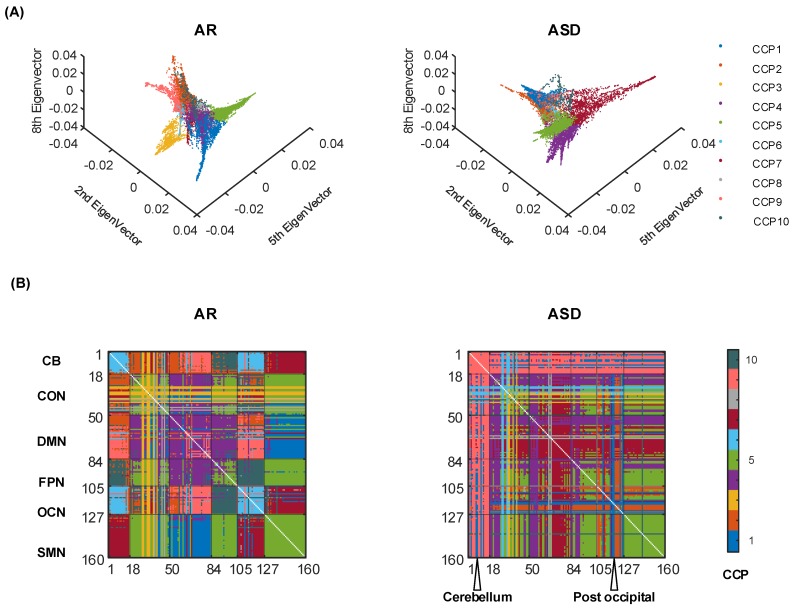
The CCPs for AR and ASD state. (**A**) The manifold structures of the CCPs for AR and ASD states are displayed in 2^nd^, 5^th^ and 8^th^ dimensions. (**B**) The CCPs in the ROI-ROI index matrix reveal the functional couplings in the networks.

Apparent changes in the CCPs under ASD and AR conditions are shown in Figure 4. One noticeable change is that in the AR condition, most ROI-based connectivity displays interleaving modularity between networks and each CCP tends to be separated into several network interactions (in Figure 4A AR subgraph). For example, CCP 5 distributes its functional connectivity between CON and SMN, FPN and SMN and the within-network interaction of these 3 networks. In the ASD condition, more CCPs turn to be ROI-leading interactions (in Figure 4B ASD subgraph). In detail, a large part of the ROIs in the cerebellum appear to connect with other ROIs that are clustered into CCP 9. Additionally, the ROIs in other networks behave with a similar trend. Another significant change is the structure of CCP organization in the manifold space. The ASD CCPs’ manifold structure becomes less dense than that of the AR state when the scatters are close to the origin. This perceptual phenomenon will be qualified in the following discussion. We estimated the matching relationship of the CCPs for the embedding representation between the resting-state of the HCP dataset and the AR group in the sleep-deprivation dataset (details shown in Table A4).

**Figure 5:**
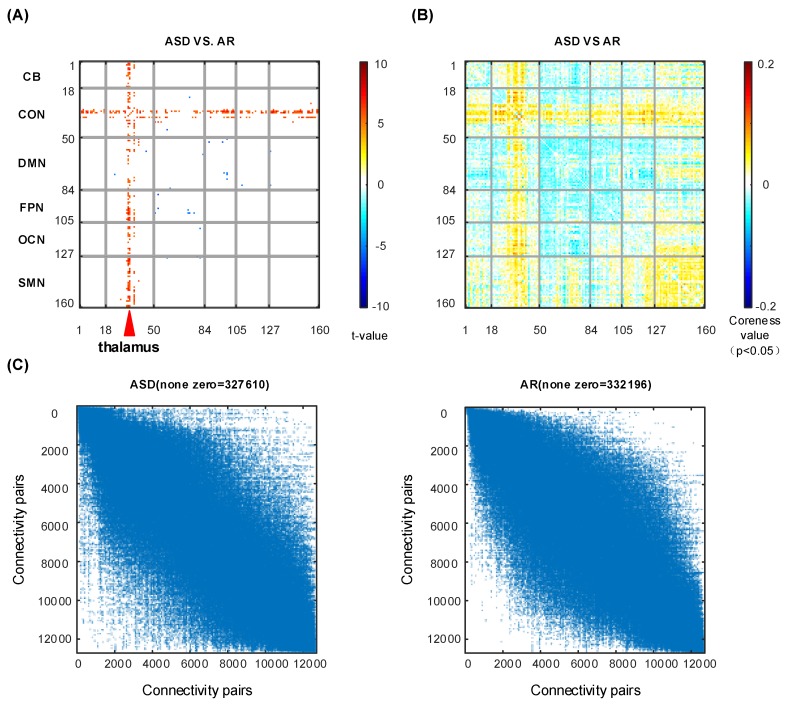
The two-sample *t*-test and permutation test for the ASD and AR states. (**A**) The two-sample *t*-test (p<0.05andFWERq<0.05) discrimination maps for ASD and AR states. (**B**) The permutation test discriminative coreness value map under p<0.05. (**C**) The adjacency matrix for ASD and AR groups. The non-zero values of the matrix are tagged on the top of the matrix.

Variations in the core-periphery structure of ASD and AR conditions at the group level were identified by using the two-sample *t*-test (p<0.05andFWERq<0.05) (shown in Figure 5). Using the core-quality values of each subject, we conducted the two-sample *t*-test using the ASD and AD labels. The experimental results can be divided into two parts (shown in Figure 5A): Most of the impact of the connectivity between thalamocortical regions with other brain regions has been more core in the ASD state; a small amount of the connectivity’s impact between the default network and occipital network, between the default network and fronto-parietal network, and between the default network and cingulo-opercular network becomes more peripheral in the ASD state. Moreover, we compare the difference in core-periphery values of ASD and AR (in Figure 5B) and the 1000 times permutation test results have given a confidence p<0.01 under the null hypothesis of exchangeability. Several ROIs (basal ganglia, thalamus, middle insula, post-insula and post-cingulate) in the cingulo-opercular network are connected with nearly all the ROIs, playing a more important role in the ASD state. Most ROIs in the sensorimotor network display a strong core influence. However, the ROIs in the default network and the fronto-parietal network tend to have more peripheral interactions with other ROIs. Then, we plotted the connectivity adjacency matrix in the ASD state, which has less of an adjacency relation than in the AR state (in Figure 5C). Furthermore, the adjacency relation’s distribution was sparser in the ASD state.

In the classification of ASD and AR, the linear SVM classifiers with a leave-one-out cross-validation strategy achieved an accuracy of 92.3% (a 10-fold cross-validation strategy achieved an accuracy of 91.6%±2.3%) when using the core-quality values as features, while a leave-one-out cross-validation strategy achieved an accuracy of 88.5% (a 10-fold cross-validation strategy achieved an accuracy of 88.0%±2.6%). If we use the original sliding-window correlation representing dynamic functional connectivity as an input feature, the linear SVM classifiers with a leave-one-out cross-validation strategy achieved an accuracy of 69.2% (a 10-fold cross-validation strategy of 65.1%±3.6%). These results indicated that using linear SVM classifiers with the core-quality values of ASD and AR structure as features could effectively distinguish the ASD and AR scans. The mean prediction accuracy 85.67% ± 3.73% of the multiple task classifier for all 6 other tasks, and the rest with a one-versus-the-rest strategy was much lower than the 7 task-rest classifiers.

## 4. Discussion

In this paper, we used local linear embedding to explore a low-dimensional manifold structure in the dynamic correlation matrix, and have successfully identified functionally homogeneous components underlying time-varying functional connectivity that was represented by a set of CCPs. These CCPs exhibited significant network-specific or subcortex-cortical coupling patterns. Importantly, we observed that the topology of the whole-brain CCPs is highly predictive for tasks or sleep-deprivation conditions relative to resting wakefulness states. These results suggested the importance of the low-dimensional representation of functional network dynamics in describing and analyzing the spatiotemporal variance of brain activities.

In the resting state, the CCP decomposition of connectivity pairs reveals significantly consistent connectivity patterns with the intrinsic connectivity networks (Figure 2B). First, the primary cortex, such as the SMN and OCN that were covered by the different single CCPs, revealed strongly functional homogeneity. The regions in the SMN were studied as a functionally separated and homogenous cortex. Researchers studied the developmental trajectory of sensorimotor cortical oscillations in the SMN-covered cortex. They would study the primary cortex as a relatively independent system to participate in some specific tasks. In contrast, the association cortex was divided into several CCPs, which was consistent with its diverse functions. These regions participate in almost all brain activities and display increased functional flexibility, potentially serving to integrate information across more specialized aspects of the cortex. In particular, the CCP 9 identified several subtle subcortex-cortical connectivities, such as the basal ganglia and the thalamus. Previous studies [39,40] have demonstrated that the cortico-basal ganglia-thalamo-cortical loop plays an important role in neural substrates of parallel processing. Our methods could extract the CCPs of the primary cortex and the association cortex, the two types of brain regions, in the connectivity pairs of the whole brain. The differentiation in the CCPs for the nonlinear manifold space (LLE), the linear low-dimensional space (PCA) and the high-dimensional Euclidean space might give evidence that the dynamic fluctuations of functional connectivity are closer to nonlinear coupling in brain activity. Because LLE recovers global nonlinear structure from locally linear fits [29]. It implies the distinction of the nonlinear functional couplings between the primary cortex and the association cortex. Although there are major differences in the scan parameters and the number, age and gender ratio of subjects, etc., the similarity of the derived components between the HCP dataset and the AR group in the sleep-deprivation condition is higher than that among single subjects in the HCP dataset. This finding might provide some information about the reproducibility and robustness of our methods. The average and standard deviation (STD) core-quality values for the core-periphery structures in the 10 CCPs show even distribution. Additionally, spatial similarity was low in the CCPs and the core-periphery structure. On the one hand, the results might partly be influenced by the fact that single-subject fMRI scans are known to be noisy to embed. On the other hand, the analyses of the obtained dFC estimation should be conducted on a group level according to previous studies [6,41]: researchers opposed the idea that similar fluctuations can arise, yielding features that can complement those of state analyses [6]. Although there are major differences in the scan parameters and the number, age and gender ratio of subjects, etc., the similarity of the derived components between the HCP dataset and the AR group in the sleep-deprivation condition is higher than that among single subjects in the HCP dataset. This finding might provide some information about the reproducibility and robustness of our methods.

Furthermore, we qualified the CCPs to detect the core-periphery structure and assigned every connectivity pair with a coreness value, where a larger value indicates a higher level of importance of a node in a graph, to explore the specific CCP configurations. Taking all matrix-mapped connectivity pairs’ coreness values into account together, we found that the core-quality value matrices showed naturally task-specific states: the resting-state connectivity pairs’ importance where the default node network inherently showed a more important impact to adapt to a task-free environment, which might occur during passive rest and mind-wandering [42]; additionally, the CCPs between the CON and whole brain show lower coreness values reflecting the weakness of the CON activity, which may be associated with the decrease in self-psychological activity during tasks. Additionally, the task-specific states are consistent with behavior in the task paradigm. In the motor task, for example, participants are required with visual cues either to tap their left or right fingers, squeeze their left or right toes, or move their tongue to map motor areas. Consequently, the motor “activation” maps present the large-scale connectivity between the sensorimotor and other brain regions to adapt to the various requirements in the specific tasks. Hence, CCPs provide a new core-quality language to describe the spatiotemporal patterns of neural activity and give a perspective to explore brain dynamics across different tasks. As validation of our approach, classification of the resting state and the tasks was designed. The accuracy of seven classifiers showing 100% exemplifies that the low manifold structures can provide behaviorally relevant information at the single-participant level. This means that the CCPs can be predicted to belong to their respective tasks. The 100% accuracy of the seven classifiers exemplifies that the potential power of the low manifold structures can provide behaviorally relevant information at the single-participant level. The core-periphery structure of the CCPs can be used to predict the respective belonging task. However, the use of the same sliding-window approach and a different block cycle in the controlled fMRI paradigm structures could influence the distinguishability between rest and tasks. Critically, the distributions of the discriminative connections are consistent with the behaviors of the tasks.

Lastly, apart from the task-dominant CCP analysis, to explore the potential capacity of our framework, we accessed the sleep-deprivation dataset of CCP changes with statistical analysis. The results reveal that the CCPs can be obviously changed. The CCPs in the AR state show consistent resting-state-like coupling patterns. Apparently different from the CCPs in the AR state where the CCPs are highly above network configurations, the ASD-induced CCPs reveal more specific region-whole-brain functional coupling peculiarity. Additionally, the increased thalamus-cortical connectivity in the two-sample *t*-test map and the discriminative regions of the cerebellum, thalamus, sensorimotor network, and default mode network in the permutation test map are in line with the previous findings [24,43,44]. Our qualification of CCP structure via statistical analysis, interestingly, reveals the discriminative regions for ASD and AR with a two-sample *t*-test (p<0.05andFWERq<0.05) at an individual level and a permutation test (p<0.05) at the group level (Figure 5). Furthermore, the nonlinear information distilled by our approach can be used to predict the participants’ internal physiological states in the ASD and AR classification task at a high-accuracy 92.3%, higher than our previous report of 88.6% accuracy [24]. This observation suggested better discriminative ability of the CCP-based classification model for the biological identity inner physiological states. Consequently, the CCPs can provide a novel description of the inner physiological state-changing population.

Besides these findings, our approach provides a new nonlinear functional coupling perspective to fMRI analysis. We introduce manifold learning of local linear embedding to explore the CCPs which reveal a functionally homogeneous region underlying dFC throughout the entire scanning. How does our brain dynamically adapt to perform different external tasks or to internal physiological status? In previous work, the brain’s discriminative functional couplings in the resting state or in the different tasks have been associated with gender [13] where the researchers used temporal-independent component analysis(tICA) methods to find a small set of connectivity patterns, and task types [11] where the investigators proposed to represent dFC as a linear combination of multiple FC patterns using principal component analysis(PCA). The gender-associated connectivity pattern work [13] is based on a hypothesis that the connectivity patterns are temporally independent in the Euclidean space. However, ICA does not have a natural ability to infer a set of subjects because different individuals will have different time courses that will be sorted differently [23]. The subject-driven task research applying PCA to estimate the FC patterns of three different tasks is based on the orthogonal decomposition of dFC, and then transfers the dFC into a linear combination of matrix eigenvectors in the Euclidean space. In contrast to these the previous works, our study focuses on the nonlinear component in the low-dimensional space that was used when analyzing fMRI data [17,18]. Moreover, by using nonlinear information and the core-quality values of CCPs, we efficiently predicted the sleep-deprivation states and task types (Table 1). We emphasize the underlying low-dimensional manifold structure in the high spatiotemporal fMRI data and uncover the CCPs of dFC in the resting state and tasks.

By using the core-quality values of CCPs and the nonlinear information extracted from our framework, we efficiently predicted the sleep-deprivation states and task types (Table 1). To exemplify the efficiency of the manifold embedding, or to determine the forecasting validity of the nonlinear information, we also used the naive connectivity matrix, e.g., from a Pearson correlation, to calculate the adjacency matrix in core-periphery structure detection. The values of the ACC, SS and SC are higher when using the embedded representation. Using the resting-state, task-based and SD fMRI data, our method could reveal CCPs in the whole-brain activity patterns and be more useful in predicting the sleep-deprivation states and task types. The input feature of the core-quality values of our approach accelerates the improvement of prediction accuracy. It can be argued that the nonlinear information of the manifold structure could be useful to improve complementary dFC methods (e.g., tICA and PCA) for examination of brain dynamics using nonlinear and linear methods. In particular, the differentiated classification accuracy rate between linear (PCA) and nonlinear (LLE) dimensionality reduction methods in method framework might imply that nonlinear structures were more able to capture the difference between the tasks and rest in the fMRI data. The prediction for the multiple tasks’ classification is more complex and changeable. The performance of the multiple task’s classifier is not better than the 7 task-rest classifiers. Furthermore, in the previous studies, in manifold decoding for neural representations of face viewpoint and gaze direction [19] and nonlinear manifold learning for connectopic mapping [45], the researchers find the manifold embedding structures were more consistent with meaningful attributes in face viewpoint, gaze direction and brain activity, rather than the extracted linear structures.

Our approach could provide two novel insights, the functionally consistent couplings in the nonlinear embedding representation and the task-specific or state-specific core-periphery structure in the manifold space, which were validated using additional fMRI analysis. Unsupervised learning (K-means algorithm) prompted the finding of functionally consistent coupling patterns (CCPs) in the manifold space. This inspired the perspective in nonlinear couplings of the connectivity pairs. The high performance of supervised classification (SVM) certified the application potential of the nonlinear structure and our framework in fMRI data. There is no doubt that the use of supervised classification increases the practical applicability of our method and the embedding representation. Furthermore, the highly distinguishable connectivity pairs for the classifiers proved that the nonlinear structure we extracted has physiological significance.

## 5. Limitation

It is vital to understand the relationship between the neural basis of cognition and our study. Our results indicate that both the CCPs and their core-periphery structure exhibit significant task effects and ASD effects (Figure 3 and Figure 4). This finding suggests that the manifold structure of the dFC can increase the sensitivity of the analysis to group effects. However, the deeper physiological significance underlying the CCPs may need more specific experiments. The methodology in our approach has some optimizable details such as parameter impact and multifrequency brain dynamics. Whether the same sliding-window approach is applicable to the task of the controlled fMRI paradigm. These analyses need further study.

## 6. Conclusions

This article presents a manifold learning of local linear embedding to distill the CCPs that reflect functionally homogeneous regions underlying the temporal evolution of dFC throughout the entire scanning period. Our results demonstrate these stable patterns of functional couplings across regions by uncovering the significant neurophysiological meaning underpinning the CCPs. The specific subcortex-cortical circuit is captured by the CCPs. Moreover, the topological organization of these low-dimensional structures exhibits high potential in predicting sleep-deprivation states (classification accuracy of 92.3%) and task types (100% identification for all seven tasks). Thus, these results suggest that our methodology for distilling coherent low-dimensional functional connectivity structures in complex brain dynamics is capable and competent.

## Figures and Tables

**Table 1:** The eight linear SVM classifiers’ accuracy with a leave-one-out cross-validation strategy for the seven tasks and the resting state, the ASD’s and AR’s resting states with the manifold structure. Sensitivity accuracy is the accuracy of emotion, gambling, language, motor, relational, social, and WM tasks and the ASD resting state. The specificity accuracy is the accuracy of the resting state and AR resting state.

Features	Task	Accuracy (%)	Sensitivity (%)	Specificity (%)
The core-quality values in manifold structure	Emotion-Rest	100	100	100
	Gambling-Rest	100	100	100
	Language-Rest	100	100	100
	Motor-Rest	100	100	100
	Relational-Rest	100	100	100
	Social-Rest	100	100	100
	WM-Rest	100	100	100
	ASD-AR	92.3	100	84.6

**Table 2:** The eight linear SVM classifiers’ accuracy with a leave-one-out cross-validation strategy for the seven tasks and the resting state, the ASD and AR resting states with the naive dFC structure. Sensitivity accuracy is the accuracy of emotion, gambling, language, motor, relational, social, and WM tasks and the ASD resting state. The specificity accuracy is the accuracy of the resting state and AR resting state.

Features	Task	Accuracy (%)	Sensitivity (%)	Specificity (%)
The core-quality values in Dynamic FC structure	Emotion-Rest	100	100	100
	Gambling-Rest	97.5	95	100
	Language-Rest	97.5	95	100
	Motor-Rest	92.5	85	100
	Relational-Rest	97.5	95	100
	Social-Rest	100	100	100
	WM-Rest	92.5	85	100
	ASD-AR	69.2	65.3	73.1

## Appendix G

**Table A1:** Labels and coordinates of the 160 ROIs.

Network	Index	ROI Label	ROI Centroid (MNI)		
			x	y	z
**cerebellum**	1	lat_cerebellum	−28	−44	−25
	2	lat_cerebellum	−24	−54	−21
	3	inf_cerebellum	−37	−54	−37
	4	lat_cerebellum	−34	−57	−24
	5	med_cerebellum	−6	−60	−15
	6	inf_cerebellum	−25	−60	−34
	7	inf_cerebellum	32	−61	−31
	8	med_cerebellum	−16	−64	−21
	9	lat_cerebellum	21	−64	−22
	10	med_cerebellum	1	−66	−24
	11	inf_cerebellum	−34	−67	−29
	12	med_cerebellum	−11	−72	−14
	13	inf_cerebellum	33	−73	−30
	14	med_cerebellum	5	−75	−11
	15	med_cerebellum	14	−75	−21
	16	inf_cerebellum	−21	−79	−33
	17	inf_cerebellum	−6	−79	−33
	18	inf_cerebellum	18	−81	−33
**cingulo-opercular**	19	aPFC	27	49	26
	20	vPFC	34	32	7
	21	ACC	−2	30	27
	22	vFC	51	23	8
	23	ant_insula	38	21	−1
	24	dACC	9	20	34
	25	ant_insula	−36	18	2
	26	basal_ganglia	−6	17	34
	27	mFC	0	15	45
	28	vFC	−46	10	14
	29	basal_ganglia	−20	6	7
	30	basal_ganglia	14	6	7
	31	vFC	−48	6	1
	32	mid_insula	37	−2	−3
	33	thalamus	−12	−3	13
	34	thalamus	−12	−12	6
	35	thalamus	11	−12	6
	36	mid_insula	32	−12	2
	37	mid_insula	−30	−14	1
	38	basal_ganglia	11	−24	2
	39	post_insula	−30	−28	−9
	40	temporal	51	−30	5
	41	post_cingulate	−4	−31	−4
	42	fusiform	54	−31	−18
	43	precuneus	8	−40	50
	44	parietal	58	−41	20
	45	temporal	43	−43	8
	46	parietal	−55	−44	30
	47	sup_temporal	42	−46	21
	48	angular_gyrus	−41	−47	29
	49	temporal	−59	−47	11
	50	TPJ	−52	−63	15
**default**	51	vmPFC	6	64	3
	52	mPFC	0	51	32
	53	aPFC	−25	51	27
	54	vmPFC	9	51	16
	55	vmPFC	−6	50	−1
	56	vmPFC	−11	45	17
	57	vmPFC	8	42	−5
	58	ACC	9	39	20
	59	vlPFC	46	39	−15
	60	sup_frontal	23	33	47
	61	sup_frontal	−16	29	54
	62	inf_temporal	52	−15	−13
	63	inf_temporal	−59	−25	−15
	64	post_cingulate	1	−26	31
	65	fusiform	28	−37	−15
	66	precuneus	−3	−38	45
	67	post_cingulate	−8	−41	3
	68	inf_temporal	−61	−41	−2
	69	occipital	−28	−42	−11
	70	post_cingulate	−5	−43	25
	71	precuneus	9	−43	25
	72	precuneus	5	−50	33
	73	post_cingulate	−5	−52	17
	74	post_cingulate	10	−55	17
	75	precuneus	−6	−56	29
	76	post_cingulate	−11	−58	17
	77	angular_gyrus	51	−59	34
	78	angular_gyrus	−48	−63	35
	79	precuneus	11	−68	42
	80	IPS	−36	−69	40
	81	occipital	−9	−72	41
	82	occipital	45	−72	29
	83	occipital	−2	−75	32
	84	occipital	−42	−76	26
**fronto-parietal**	85	aPFC	29	57	18
	86	aPFC	−29	57	10
	87	vent_aPFC	42	48	−3
	88	vent_aPFC	−43	47	2
	89	vlPFC	39	42	16
	90	dlPFC	40	36	29
	91	ACC	−1	28	40
	92	dlPFC	46	28	31
	93	vPFC	−52	28	17
	94	dlPFC	−44	27	33
	95	dFC	40	17	40
	96	dFC	44	8	34
	97	dFC	−42	7	36
	98	IPL	−41	−40	42
	99	IPL	54	−44	43
	100	post_parietal	−35	−46	48
	101	IPL	−48	−47	49
	102	IPL	−53	−50	39
	103	IPL	44	−52	47
	104	IPS	−32	−58	46
	105	IPS	32	−59	41
**occipital**	106	occipital	−18	−50	1
	107	occipital	−34	−60	−5
	108	occipital	36	−60	−8
	109	temporal	46	−62	5
	110	occipital	−44	−63	−7
	111	occipital	19	−66	−1
	112	occipital	17	−68	20
	113	occipital	39	−71	13
	114	occipital	29	−73	29
	115	occipital	−29	−75	28
	116	occipital	−16	−76	33
	117	occipital	9	−76	14
	118	occipital	15	−77	32
	119	occipital	20	−78	−2
	120	post_occipital	−5	−80	9
	121	post_occipital	29	−81	14
	122	post_occipital	33	−81	−2
	123	post_occipital	−37	−83	−2
	124	post_occipital	−29	−88	8
	125	post_occipital	13	−91	2
	126	post_occipital	27	−91	2
	127	post_occipital	−4	−94	12
**sensorimotor**	128	frontal	58	11	14
	129	dFC	60	8	34
	130	vFC	−55	7	23
	131	pre_SMA	10	5	51
	132	vFC	43	1	12
	133	SMA	0	−1	52
	134	frontal	53	−3	32
	135	precentral_gyrus	58	−3	17
	136	mid_insula	−42	−3	11
	137	precentral_gyrus	−44	−6	49
	138	parietal	−26	−8	54
	139	precentral_gyrus	46	−8	24
	140	precentral_gyrus	−54	−9	23
	141	precentral_gyrus	44	−11	38
	142	parietal	−47	−12	36
	143	mid_insula	33	−12	16
	144	mid_insula	−36	−12	15
	145	temporal	59	−13	8
	146	parietal	−38	−15	59
	147	parietal	−47	−18	50
	148	parietal	46	−20	45
	149	parietal	−55	−22	38
	150	precentral_gyrus	−54	−22	22
	151	temporal	−54	−22	9
	152	parietal	41	−23	55
	153	post_insula	42	−24	17
	154	parietal	18	−27	62
	155	parietal	−38	−27	60
	156	parietal	−24	−30	64
	157	post_parietal	−41	−31	48
	158	temporal	−41	−37	16
	159	temporal	−53	−37	13
	160	sup_parietal	34	−39	65

## Appendix H

**Table A2:** The SOR value between the CCPs of the subject 1 and subject 2 in the manifold space.

CCP (%)	1	2	3	4	5	6	7	8	9	10
1	3.38	4.84	5.84	6.16	7.53	2.11	8.65	3.28	5.54	7.19
2	3.33	6.66	5.17	3.60	4.82	3.82	7.52	5.13	3.44	7.25
3	4.13	4.14	4.75	5.97	5.42	5.95	5.12	4.74	6.31	5.90
4	5.36	6.02	5.19	3.94	5.07	6.99	5.17	5.95	7.15	4.55
5	3.38	3.83	4.14	1.79	3.54	1.37	7.78	5.74	6.77	8.08
6	13.39	3.56	5.40	0.58	2.66	9.91	4.12	6.18	1.98	4.01
7	6.24	5.56	5.71	2.23	7.76	5.71	3.75	4.69	5.30	5.49
8	9.28	4.93	5.94	1.48	3.01	7.64	2.67	6.47	5.42	4.92
9	6.86	5.97	4.51	4.47	5.92	5.55	5.09	5.32	5.09	6.32
10	4.22	3.25	7.17	4.59	5.71	5.19	5.47	4.59	6.02	5.64

## Appendix I

**Table A3:** The $eta^{2}$ similarity for the core-periphery structure among the first 10 subjects of the HCP resting-state dataset.

${\mathrm{eta}}^{2}$ (%)	sub2	sub3	sub4	sub5	sub6	sub7	sub8	sub9	sub10
sub1	51.13	54.13	56.65	58.20	54.72	53.55	52.57	57.94	53.17
sub2		48.34	52.29	50.60	50.97	52.14	54.95	54.59	50.15
sub3			58.53	59.93	55.68	56.38	51.98	55.12	61.19
sub4				61.35	55.68	59.29	53.58	59.11	58.70
sub5					56.72	55.54	53.83	61.31	60.09
sub6						53.89	52.91	55.94	56.15
sub7							53.93	54.69	57.72
sub8								51.31	53.50
sub9									55.59

## Appendix J

**Table A4:** The SOR value for the embedding representation between the resting state of the HCP dataset and the AR group in the sleep-deprivation condition.

CCP (%)	1	2	3	4	5	6	7	8	9	10
1	6.90	1.01	0.07	1.63	46.18	0.00	0.15	1.88	3.17	0.00
2	3.92	7.22	14.12	3.72	1.26	4.42	0.00	5.93	0.00	0.00
3	2.21	5.81	5.31	0.80	2.33	0.94	0.00	0.49	43.48	0.94
4	0.22	7.13	30.45	1.28	6.02	0.00	0.42	17.91	0.11	1.54
5	0.48	6.11	1.58	38.52	4.18	0.00	24.21	0.17	4.39	2.17
6	14.21	7.83	2.27	0.53	0.09	7.74	1.02	3.10	0.00	0.00
7	25.39	9.83	0.59	5.66	0.55	4.75	0.00	2.22	3.44	0.00
8	0.77	0.04	0.00	0.54	0.10	0.37	0.08	0.05	0.22	69.12
9	3.14	0.70	5.65	0.00	4.80	3.60	0.06	28.43	0.00	0.00
10	12.13	15.45	4.40	7.30	0.94	3.01	0.00	4.44	0.00	2.08

## Appendix K

**Table A5:** The average and standard deviation (STD) core-quality values for the core-periphery structures in the 10 CCPs.

The Core-Quality Value	1	2	3	4	5	6	7	8	9	10
mean	0.63	0.61	0.61	0.62	0.62	0.64	0.63	0.61	0.61	0.59
STD	0.01	0.01	0.01	0.01	0.02	0.02	0.02	0.01	0.02	0.10

## Appendix L

**Table A6:** The prediction accuracy of the classifier based on the linear-dimensional (PCA) framework.

Accuracy (%)	Emotion	Gambling	Language	Motor	Relational	Social	WM
Resting-state	86.25	91.00	92.75	83.25	95.75	96.75	83.50
